# North Atlantic Blue and Fin Whales Suspend Their Spring Migration to Forage in Middle Latitudes: Building up Energy Reserves for the Journey?

**DOI:** 10.1371/journal.pone.0076507

**Published:** 2013-10-08

**Authors:** Mónica A. Silva, Rui Prieto, Ian Jonsen, Mark F. Baumgartner, Ricardo S. Santos

**Affiliations:** 1 Center of the Institute of Marine Research (IMAR) & Department of Oceanography and Fisheries, University of the Azores, Horta, Azores, Portugal; 2 Laboratory of Robotics and Systems in Engineering and Science (LARSyS), Lisboa, Portugal; 3 Biology Department, Woods Hole Oceanographic Institution, Woods Hole, Massachusetts, United States of America; 4 Department of Biology, Dalhousie University, Halifax, Nova Scotia, Canada; CSIR- National institute of oceanography, India

## Abstract

The need to balance energy reserves during migration is a critical factor for most long-distance migrants and an important determinant of migratory strategies in birds, insects and land mammals. Large baleen whales migrate annually between foraging and breeding sites, crossing vast ocean areas where food is seldom abundant. How whales respond to the demands and constraints of such long migrations remains unknown. We applied a behaviour discriminating hierarchical state-space model to the satellite tracking data of 12 fin whales and 3 blue whales tagged off the Azores, to investigate their movements, behaviour (transiting and area-restricted search, ARS) and daily activity cycles during the spring migration. Fin and blue whales remained at middle latitudes for prolonged periods, spending most of their time there in ARS behaviour. While near the Azores, fin whale ARS behaviour occurred within a restricted area, with a high degree of overlap among whales. There were noticeable behavioural differences along the migratory pathway of fin whales tracked to higher latitudes: ARS occurred only in the Azores and north of 56°N, whereas in between these areas whales travelled at higher overall speeds while maintaining a nearly direct trajectory. This suggests fin whales may alternate periods of active migration with periods of extended use of specific habitats along the migratory route. ARS behaviour in blue whales occurred over a much wider area as whales slowly progressed northwards. The tracks of these whales terminated still at middle latitudes, before any behavioural switch was detected. Fin whales exhibited behavioural-specific diel rhythms in swimming speed but these varied significantly between geographic areas, possibly due to differences in the day-night cycle across areas. Finally, we show a link between fin whales seen in the Azores and those summering in eastern Greenland-western Iceland along a migratory corridor located in central Atlantic waters.

## Introduction

Most baleen whales are thought to migrate every year between high-latitude productive areas, where they spend the summer feeding, to tropical or sub-tropical oligotrophic wintering grounds used for mating and calving. While the selective pressures behind the evolution of baleen whale migration are still a matter of discussion (e.g. [1]–[5]), the mere fact that whales must devote substantial amounts of time and energy each year moving between widely separated geographic areas, makes migration an important life-history component of these whales. More importantly, periods of the annual cycle are inextricably linked such that ecological circumstances within one season and life stage are likely to affect animals’ behaviour, performance, and even survival in later stages [6]. For example, food availability during the foraging season can determine both timing of migration and physical condition at departure, which in turn can influence arrival time and physical condition at the breeding ground, ultimately affecting reproductive success. Occurrence of bad weather conditions can delay reproduction and subsequent arrival at the feeding areas, creating the potential for a temporal mismatch between food demand and supply, or forcing animals to occupy lower-quality habitats [7]. Ecological conditions encountered during feeding and breeding seasons are known to be a primary driver of population dynamics in a wide range of taxa, but evidence is accumulating that conditions experienced during migration may be equally decisive [7]. Despite its obvious importance, it’s remarkable how little we know about the migratory behaviour and strategies of most baleen whales.

During the period of intensive feeding activity at the summer habitats, large baleen whales acquire substantial quantities of energy reserves and store the surplus of that energy in the form of fat depots [8]. It is believed that whales feed only opportunistically and at reduced levels in their migratory and wintering habitats and fat stored during the summer feeding season largely finances reproduction, as well as the south- and northbound migratory journeys [1], [3], [4], [8]. Multiple lines of evidence support the “feast and famine” hypothesis of baleen whale migration: i) food availability outside polar and subpolar feeding grounds is generally much lower and believed to be insufficient to satisfy prey requirements of large whales; ii) whales caught at the wintering and migratory grounds often had empty stomachs or had consumed only small amounts of food [4]; and iii) theoretical physiological models indicated that the larger body size of whales allowed them to endure longer periods of fasting [9]. Recent kinematic studies of foraging behaviour of balaenopterid whales also provide indirect support to this hypothesis: the high energetic costs associated with lunge feeding behaviour likely impose a foraging threshold, thereby limiting feeding activity to areas of very high prey density [10]–[12].

Nevertheless, feeding in mid- to low-latitude waters can occur in some species. At sea observations and satellite tracking studies of humpback whales (*Megaptera novaeangliae*) revealed that some whales can pause their migratory journey to the summer grounds to forage in middle latitude areas and even at the wintering grounds [13]–[15]. While knowledge of migratory tactics is far more limited for other rorquals, a recent study of satellite-monitored movements of blue whales (*Balaenoptera musculus*) in the Northeast Pacific reported occurrence of area-restricted search (ARS [16]; see below) behaviours throughout their migratory cycle and at different latitudes [17]. This finding suggests the possibility that blue whales forage year-round, substantiating earlier assumptions based on sighting data and on a small number of tagged individuals [18], [19]. In the Northeast Atlantic, blue and sei whales (*B. borealis*) were observed feeding outside their typical foraging grounds (e.g. Madeira (20°N), Mid-Atlantic Ridge (40–55°N) and on Porcupine Sea Bight and Banks (52–53°N); [20]–[22]) but such reports are rare and scattered in time. Thus, it remains unclear how widespread this tactic may be among populations and whether it is adopted by other long-distance migrants such as the fin whale (*B. physalus*).

The Azores offers an excellent opportunity to investigate the migratory strategies of North Atlantic blue and fin whales. Autumn and winter sightings of these species are rare [23] but both are commonly seen every year in spring and summer, presumably as they migrate through the area on the way to the northern feeding grounds [23], [24]. Blue and fin whales are known to feed in the vicinity of the Azorean islands [24], [25] but we still don’t know how long individual whales stay at this migratory habitat, what are their daily movements and activities in the region, and whether they feed at other sites along their path. Answering these questions is a first step to understanding the relevance of feeding at migratory habitats for large whales, and whether and how this strategy may cascade through later stages of their life cycle.

In this paper we present the results of a study that explores the movements and behaviour of North Atlantic blue and fin whales during their northward spring migration, using tracks from whales instrumented with satellite transmitters in the Azores. We applied a Bayesian hierarchical switching state-space model (hSSSM) [26], [27] to the tracking data to obtain improved estimates of whale locations and movement parameters from Argos-derived positions and to infer migratory (fast, directed movements) and ARS behaviours within tracks. Animals feeding on patchily distributed resources are expected to engage in ARS (by increasing turning angles and decreasing travel rates) when encountering sufficiently abundant prey, to increase search effort in the most profitable areas [28]. We then used data derived from the models to investigate: (1) evidence of foraging at middle latitudes; (2) diel, monthly and interannual differences in whales’ movements and behaviour; (3) migratory routes and destinations; and (4) activity patterns along the migratory journey.

## Materials and Methods

### Ethics Statement

Fieldwork and tagging were approved by the relevant authorities (Regional Directorate for Sea Affairs, Autonomous Region of the Azores) under research permits: 20/2009/DRA, 16/2010/DRA, 51/2011/DRA, 31/2012/DRA. All procedures followed the guidelines of the American Society of Mammalogists [29].

### Data Collection

**Figure 1:**
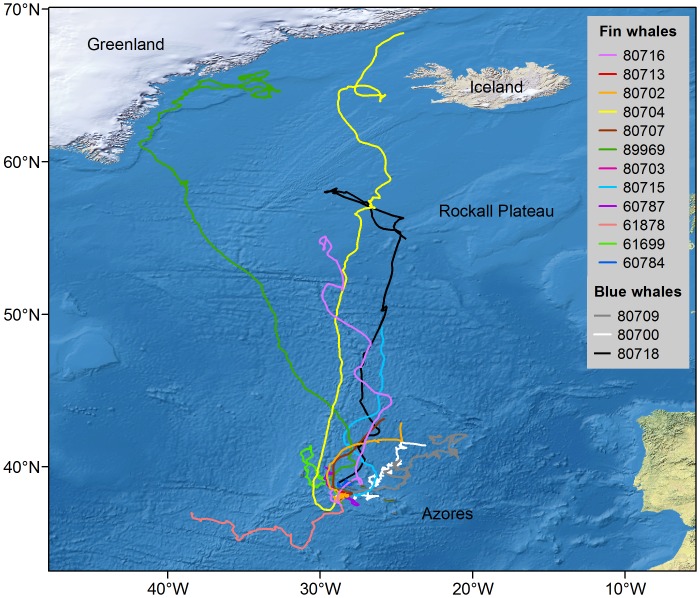
Hierarchical switching state-space model derived tracks of 12 fin whales and 3 blue whales.

Satellite-linked radio transmitter tags were attached to 12 fin whales and 4 blue whales as they migrated through the Archipelago of the Azores, Portugal. Whales were tagged off Faial and Pico islands (38°N 28°W, Fig. 1) from March to May 2009 to 2012, except one fin whale tagged in September 2009 (Table 1). The transmitters (model SPOT5-implantable, Wildlife Computers, Redmond, Washington, USA) were housed in stainless-steel cylinders that were attached to the whales’ back with a four-bladed point and held in place with 4 sets of barbs and 6 backward-facing petals. Tags were surgically sterilized and the anchoring system was coated with Gentamicyn sulfate antibiotic prior to implantation. Tags were deployed from a 6-m rigid-hulled inflatable or a 12-m fiberglass boat using a compressed air gun (ARTS/RN, Restech Norway) set at 8–10 bar pressure. The tags were attached anterior to the dorsal fin of the whales. All tags were programmed to transmit on a daily basis, every hour of the day up to a maximum of 500 messages per day.

**Table 1:** Tracking data for fin and blue whales satellite-tagged off Azores from 2009 to 2012.

Species	WhaleId	AgeClass*	Tagging date	Track duration (days)	N° locations received	Mean n°locations/day	Mean timestep (hour)	Distance travelled (km)
Fin whales	80716	Ad	01/09/2009	18	119	6.6	3.5	2,920
	80713	Ad	17/04/2010	3	30	10.0	1.5	98
	80702	Ad	30/04/2010	18	253	14.1	1.6	1,664
	80707	Ad	30/04/2010	19	256	13.5	1.7	1,537
	80704	Ad	12/05/2010	34	335	9.9	2.4	4,584
	89969	Ad	12/05/2010	55	2127	38.7	0.6	6,019
	80703	Ad	26/04/2011	2	15	7.5	1.5	32
	80715	Ad	23/05/2011	14	90	6.4	3.5	1,674
	60787	Ad	15/03/2012	11	139	12.6	1.8	780
	61878	Ad	16/03/2012	17	210	12.4	1.9	1,741
	61699		04/04/2012	21	353	16.8	1.3	1,493
	60784	Juv	20/04/2012	11	109	9.9	2.2	628
Blue whales	80709	Ad	27/04/2009	60	353	5.9	4.0	3,063
	80700	Ad	06/05/2009	45	449	10.0	2.4	2,315
	80718	Ad	20/05/2011	22	125	5.7	4.1	3,073

* Ad: Adult; Juv: Juvenile.

### Switching State-space Model

We fitted the Bayesian switching state-space model (SSSM) described in Jonsen et al. [30] to Argos-acquired satellite locations to analyse movements and behaviour of tagged whales. Location data were derived from the new positioning algorithm implemented by Argos that accounts for movement dynamics and uses a Kalman filter (KF) to calculate positions [31], [32]. The new algorithm is reported to increase the number of estimated positions and improve their accuracy [32].

State-space models couple two stochastic models: a process model (transition equation) that predicts the future state, the location and behavioural state, of an animal given its current state, and an observation model that relates the unobserved location states predicted by the process model to the observed data (locations obtained from Argos). The SSSM uses a first-difference correlated random walk as the process model to describe movement dynamics [30] but allows movement parameters to change between two discrete behavioural states by including a process model for each one.

In the present study, the SSSM was fit as a single hierarchical model to all tracks simultaneously within each species [27]. Hierarchical switching state-space models (hSSSM) have the advantage of combining information from all tracking data to estimate parameters at both the individual and population levels, leading to more efficient parameter estimation [33]. In addition, these models enable parameter estimation even for shorter or incomplete tracks, by analysing these together with data from other tracks. Thus, by letting *k* index each individual whale, the transition equation specified in Jonsen et al. [34] formulated within a hierarchical framework becomes:
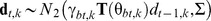
where **d** *_t_*_-1,*k*_ is the displacement of whale *k* between unobserved locations **x** *_t_*_-1_ and **x** *_t_*_-2,_ and **d** *_t,k_* is the displacement of whale *k* between unobserved locations **x** *_t_* and **x** *_t_*_-1_. **T**(θ) is a transition matrix that provides the rotation required to move from **d** *_t_*_-1_ to **d** *_t_*, where θ is the mean turning angle. γ is the move persistence coefficient (i.e. combined autocorrelation in direction and speed). *N*_2_ is a bivariate Gaussian distribution with covariance matrix Σ and represents the randomness in animal movement. The movement parameters θ and γ are indexed by behavioural state *bt*. At each displacement *t* of whale *k*, the estimated behavioural state *b* corresponds to the set of parameters θ and γ that provide the best model fit. We placed the same priors on movement parameters as Breed et al. [35], assuming that during transiting turn angles should be close to 0° and autocorrelation in speed and direction should be higher than during ARS.

The observation model accounts for the irregularity and variable errors in the observed Argos locations. Errors in latitude and longitude are modelled with a *t*-distribution using independent parameter estimates derived for each Argos location class [30], [36].

The hSSSM was fit using a time step of 2 h for fin whales and 4 h for blue whales (comprising 90% of time steps recorded for each species). These time steps allowed us to examine movements and behaviour of these whales at finer scales, infer short ARS bouts and the approximate timing of behavioural switches. Models were fit using R (R Development Core Team 2008) code provided in the supplement to Jonsen et al. [27]. The code implements the hSSSM using Markov Chain Monte Carlo (MCMC) methods via JAGS. For each hSSSM we run two MCMC chains for 50000 iterations, dropping the first 45000 samples as a burn-in and retaining every 5^th^ sample from the remaining 5000 assumed post-convergence samples to reduce sample autocorrelation. Thus, model parameters and estimates of whales’ locations and behaviours were calculated using a total of 2000 MCMC samples. Model convergence and sample autocorrelation were assessed by visually inspecting trace and autocorrelation plots and using the Gelman and Rubin diagnostic available in R package boa.

### Analysis of Whale Tracks

Whale behaviour at each 2-h (for fin whales) or 4-h location (for blue whales) was inferred from the output of the hSSSM. Because behaviour is treated as a binary variable MCMC samples can only assume the values 1 (transiting) or 2 (ARS), *b* at each location was estimated as the mean value of the MCMC samples. We used the same cut off points as Jonsen et al. [34]: locations with mean estimates of *b*<1.25 were assumed to represent transiting, *b*>1.75 ARS, and between these values were considered “uncertain”.

For most whales, departure from the Azores was easily detected by a switch in behavioural mode, followed by a straight track and equally spaced locations. Timing and location of departure was less clear for tracks with frequent transitions in behaviour, reversals in direction and a large number of “uncertain” locations. We therefore defined timing of departure from the Azores as the first of ≥48 consecutive hours with the whale travelling at speeds higher than the median ARS speed estimated from all whale tracks.

The reduced number and limited extent of blue whale tracks prevented a detailed analysis of migratory movements and behaviour for this species. Therefore, residency and monthly variation in ARS behaviours in the Azores, and comparison of movements between geographic areas were only assessed for fin whales.

Once departure time had been established for all whales, minimum residence time of fin whales in the Azores was investigated by two methods, using data from whales tracked >10 days. The probability of whales departing from the Azores in relation to number of days since tagging (DST) was estimated through a logistic regression, with the total number of whales present each day after tagging as the response variable. To account for variability in track duration, the response variable was weighted by the total number of whales with working tags on the same relative DST of their track. Time to whale departure was also determined by fitting the non-parametric Kaplan-Meier estimator of survival probability. Survival analysis offers the advantage of accounting for censored data, when information on time to event is not available for all subjects. In this way, data from whales whose tags stopped transmitting prior to departure from the Azores could also be included in the estimation of departure probability.

Monthly variations in the proportion of tracking time fin whales spent in ARS per day in the Azores were examined using a Generalized Linear Mixed Model (GLMM) with a Binomial error distribution and a logit link function, and including individual track as a random effect.

We compared the behaviour and movements of fin whales in different geographic areas along their migratory pathway to investigate the existence of distinct migratory phases and to understand whales’ activity patterns at each phase. For each area we calculated the mean proportion of tracking time spent transiting and in ARS, swimming speed during transiting and ARS behaviours, and the number, size and time spent within discrete ARS areas. We defined a discrete ARS area as 3 or more consecutive positions within a track with *b*>1.75 [17] and calculated the size of each area using minimum convex polygons.

In addition we investigated daily rhythms in fin whales’ activity at each geographic area, by looking at the effect of time of day on the occurrence of ARS and on swimming speed during different behaviours. The effect of hour on the probability of occurrence of ARS was assessed by fitting a GLMM with a Binomial error distribution with a logit link function and including whale track as a random effect. Generalized Additive Models (GAM) with a Gaussian error distribution and an identity link function were used to investigate the effect of latitude, area, hour, and the interaction of the latter on speed during transiting and ARS. A likelihood ratio test for analysis of variance of circular data was used to investigate if turn angles between consecutive ARS locations varied among areas and throughout the day.

Effect of hour on the probability of occurrence of ARS behaviour and on ARS swimming speed in blue whales was examined using a GLMM (Binomial error distribution and logit link function, with whale track as a random effect) and GAM (Gaussian error distribution and identity link function), respectively. Circular ANOVA was used to assess hourly differences in turn angles between displacements of blue whales in ARS.

Unless otherwise stated, means are presented ± standard deviation (SD). Analyses were performed in R software using packages survival, lme4, MASS, mgcv and circular.

## Results

### Whale Tracks and Model Performance

Tracks of the 12 fin whales averaged 18 days in length with a mean of 19.0±14.9 satellite locations per day (Table 1). One of the tags deployed on a blue whale never transmitted, possibly due to tag failure. Duration of the 3 blue whale tracks ranged from 22 to 60 days with a mean of 7.3 (±4.0) locations per day (Table 1). We obtained fewer locations per day for 2 blue whales due to the imperfect placement of the satellite tag (slightly low on the whales’ flank). Mean number of locations received daily for the other blue whale was within the range of values obtained for fin whales.

The hSSSM were fit to the tracks of these 12 fin whales and 3 blue whales. Results from the diagnostic tests and inspection of lag-autocorrelation and trace plots suggest that MCMC chains of the two hSSSM models converged and provided representative samples from the posterior distributions. Models distinguished well between two behavioural modes, as indicated by the parameter estimates that aggregated into two non-overlapping groups (Table S1). Transiting behaviour was characterized by nearly linear track segments and similar intervals between consecutive displacements, whereas the second type of movement, classified as ARS, was characterized by lower persistence in speed and direction and frequent reversals (Table S1).

About 47% of a total of 2540 fin whale locations were inferred as transiting, 40% as ARS and 13% as uncertain. The hSSSM applied to blue whale tracks classified 20% and 66% of locations as transiting and ARS, respectively, and 14% as uncertain.

### Fin Whales

#### Residence, movements and behaviour at the Azores

All fin whales remained in the Azores after being tagged (Fig. 1, Table 2). Two tags stopped transmitting 2 and 3 days after being deployed while the whales were still in the vicinity of the islands. Whales tracked for >10 days stayed between 4 and 22 days in the area (Table 2).

**Table 2:** Estimates of fin and blue whale residence time and time spent in ARS in the Azores.

Species	Whale#	Tagging date	Residence time(days)	Proportion totaltrack time	Proportion track timein ARS
Fin whales	80716	01/09/2009	6	0.33	8.2
	80713*	17/04/2010	3	100	100
	80702	30/04/2010	13	0.76	92.9
	80707	30/04/2010	17	0.94	98.4
	80704	12/05/2010	6	0.18	22.2
	89969	12/05/2010	4	0.07	50.0
	80703*	26/04/2011	2	100	100
	80715	23/05/2011	3	0.23	39.1
	60787	15/03/2012	11	100	59.3
	61878	16/03/2012	18	100	13.8
	61699	04/04/2012	22	100	78.5
	60784	20/04/2012	12	100	90.2
Mean			11.2	0.65	55.3
Blue whales	80709	27/04/2009	60	100	73.5
	80700	06/05/2009	45	100	82.5
	80718	20/05/2011	0	0	
Mean			35	66.7	78.0

* Whales with short-duration tracks that were not included in the calculation of mean values of residence time and time spent in ARS.

**Figure 2:**
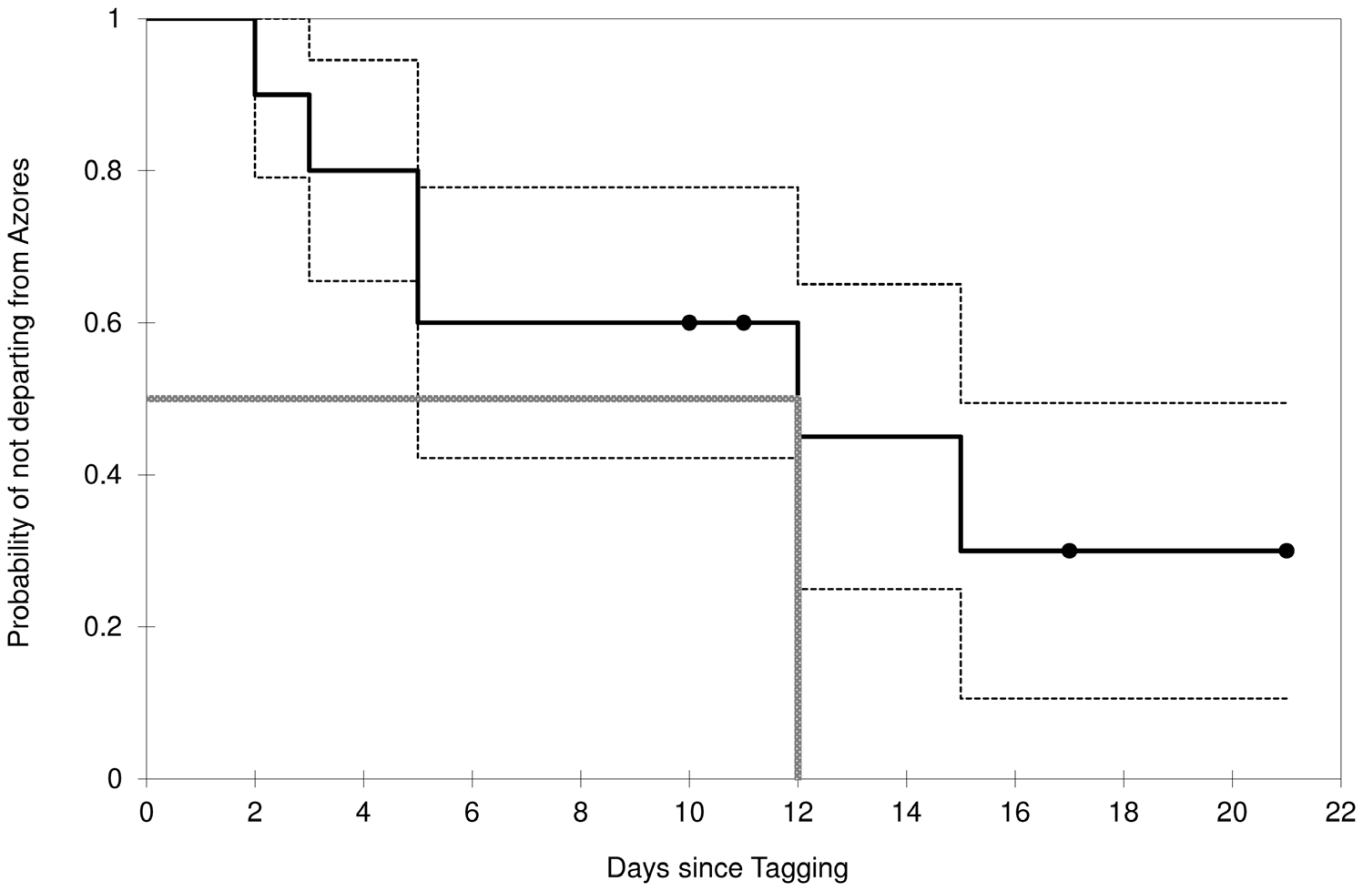
Probability of fin whales not departing from the Azores over time. Kaplan-Meier estimate (solid line) of the probability of fin whales not departing (1-probability of departure) from the Azores at various days since tagging (DST). The black dots represent the censored observations (whales whose tags stopped transmitting prior to departure). The dotted lines represent the 25% and 75% estimated probabilities of fin whales not departing from the Azores in relation to DST. The median DST to departure (the DST at which half of the whales have departed) is indicated by the gray dashed line.

Probability of whales departing from the Azores increased significantly over time (logistic regression: z = 5.448, *P*<0.001), with a 50% chance of departure predicted at 11.9 (SE = 0.97) DST. Although this value is certainly underestimated due to the premature interruption of 4 tags, it is consistent with the results from survival analysis that takes into account censored observations. The Kaplan-Meier survivor function estimated the median departure time at DST = 12, and the 25% and 75% probabilities of departure at DST = 5 and DST = 15, respectively (Fig. 2). Week of tagging affected length of residence in the Azores (exponential regression model: r = 0.64, *P* = 0.011). Whales tagged earlier (15 March–30 April) remained longer in the Azores (≥11 days) than whales tagged after week 20 that only spent 3–6 days in the area (which represented 7–33% of their total track time) (Table 2). Residence time was not correlated with proportion of time whales were in ARS (Spearman’s rank correlation: r = 0.42, t = 1.31, *P* = 0.230). All whales tagged in spring (n = 9) departed from the Azores between 12 and 25 of May. On a single occasion we observed 2 whales leaving the area nearly simultaneously though they did not travel together subsequently.

**Figure 3:**
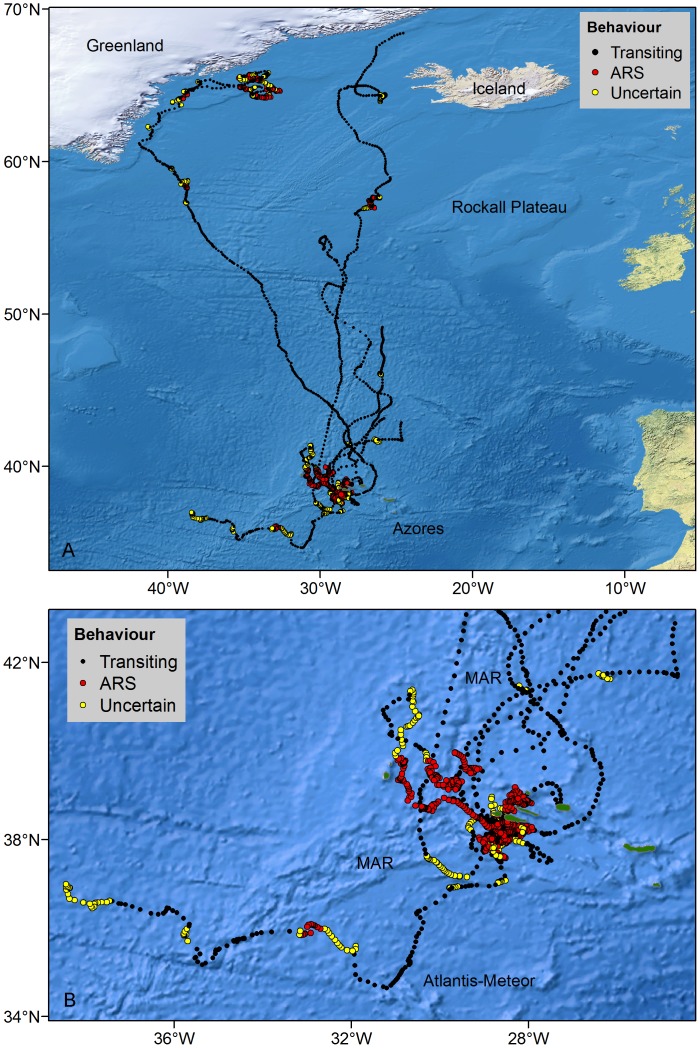
Hierarchical switching state-space model derived locations of fin whales showing inferred behavioural modes. A. Complete tracks. B. Details of the tracks at middle latitudes, showing the location of the Mid-Atlantic Ridge (MAR) and the Atlantis-Meteor seamount complex.

The model inferred ARS behaviour in the Azores in all fin whale tracks (Figs. 3A and 3B, Table 2). Whales tracked for 2 and 3 days spent 100% of their time in ARS; data from these tracks were not included in further analyses. The remaining whales spent considerable more time in ARS than in transit (Table 2). Proportion of time spent in ARS per day varied between months, being significantly higher in April (82±38%) and May (74±42%) (Table S2).

**Figure 4:**
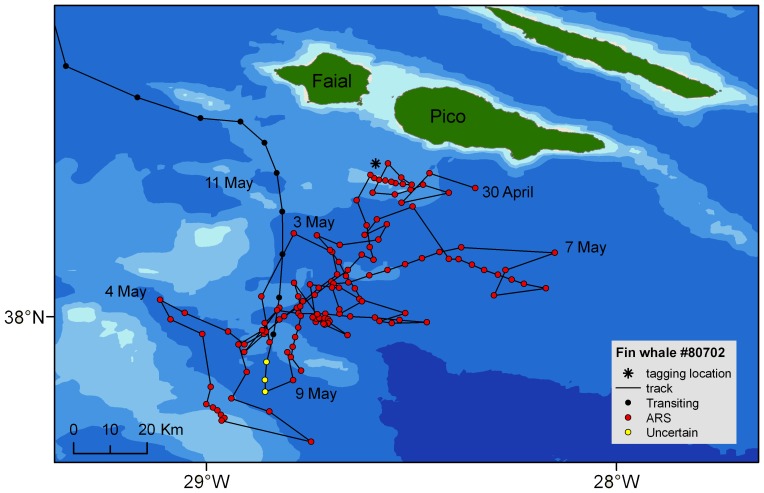
Example of the movements and behaviour of fin whales in the Azores in 2009–2011. Whale #80702 was tagged on April 30 2010 (tagging location shown by a star) and departed on 11 May 2010. The track shows the inshore-offshore movement pattern south of the islands of Faial and Pico and along the shallow banks where the whale was in ARS for 12 days, and the clear behavioural switch associated with the north/northwest trajectory as it resumed migration.

Ten discrete ARS areas were identified in the Azores, varying in size from 2 to 11,619 km^2^ (Table 3). In general, ARS areas largely overlapped and were very close to tagging locations. Distance between tagging location and the centroid of ARS areas for all whales was 77±128 km. ARS mainly occurred south of the islands of Faial and Pico and along a chain of shallow seamounts extending 90 km to the south of these islands (Fig. 3B). Movements within this area were broadly similar among whales, with individuals making repeated inshore-offshore movements between the islands and the banks (Fig. 4). These whales switched from ARS to transiting behaviour as they moved west/northwest until passing the island of Faial, after which no whale turned back. One whale tagged north of Faial remained there in ARS for 11 days. Three whales tagged in 2012 showed slightly different movement patterns (Fig. 3B). After spending 2–3 days in ARS south of Pico, whales 60787 and 61699 headed west/northwest and spent the rest of their tracking period (9 and 18 days) near the Mid-Atlantic Ridge (MAR), alternating between ARS and transiting. Whale 61878 travelled 500 km south to the Atlantis-Meteor seamount complex. Upon reaching the seamount, it turned northwest, meandering slowly towards MAR. It remained around the ridge for 5 days, 2 of which in ARS, eventually crossing to the western side of the ridge, where the track terminated 4 days later.

**Table 3:** Fin whale movements and behaviour at different stages along the migratory path.

	Stationary stage		Migratory stage
	Azores	>56°N	40–56°N
	(n = 10 whales)	(n = 2 whales)	(n = 6 whales)
Track time in transit (%)	23±39%	37±39%	100
Track time in ARS (%)	55±34%	34±41%	0
N° ARS areas	10	10	0
Size ARS areas (km^2^)	2,036±3,618	600±605	
Time (h) within ARS area	99.6±121.5	45.2±27.8	
Transiting speed (km.h^−1^)	5.7±3.2	5.7±2.5	7.7±3.8
ARS speed (km.h^−1^)	2.5±1.9	2.8±1.6	

#### Movements and behaviour along the migratory path

Tracks of 6 fin whales provided information on whales’ movements and behaviour after their departure from the Azores and revealed substantial changes along the migratory pathway (Fig. 1, Table 3). As they moved away from middle latitudes, all whales followed a generally northerly direction (heading: 32.8±0.8° angular deviation), including the individual tagged in September that was at 55°N when the track terminated. Three other tags stopped before the whales reached 50°N. Although all 6 tracks included periods of erratic movement, ARS was not detected until whales reached 57°N (Fig. 3A, Table 3).

Fin whales’ speed during transiting behaviour also changed along the migratory pathway, with evident downward peaks coincident with the range of latitudes where ARS behaviour occurred and higher speeds recorded at intermediate latitudes (Fig. S1). Mean transiting speed was considerably slower in the Azores and north of 56° than between these latitudes (Table 3, Table S3). Movements and behaviour of fin whales in the Azores and north of 56° were broadly similar but whales spent less time engaged in ARS at northern latitudes, ARS areas were smaller and whales spent less time within each ARS area (Table 3, Table S3).

Two fin whales were tracked to the presumed feeding grounds (Figs. 1 and 3A). Whale 89969 travelled 3,200 km in 21 days in a nearly direct path between the Azores and Greenland. It then spent 1 month over the Greenland eastern continental shelf and along the shelf slope, switching between ARS and transiting. Whale 80704 spent 88 hours within an ARS area 350 km northwest of the Rockall Plateau (57°N), before continuing transiting through the Irminger Sea <200 km from the western coast of Iceland and ending up over the Greenland shelf, approximately at 68°N.

#### Diel variation in movements and behavior

**Figure 5:**
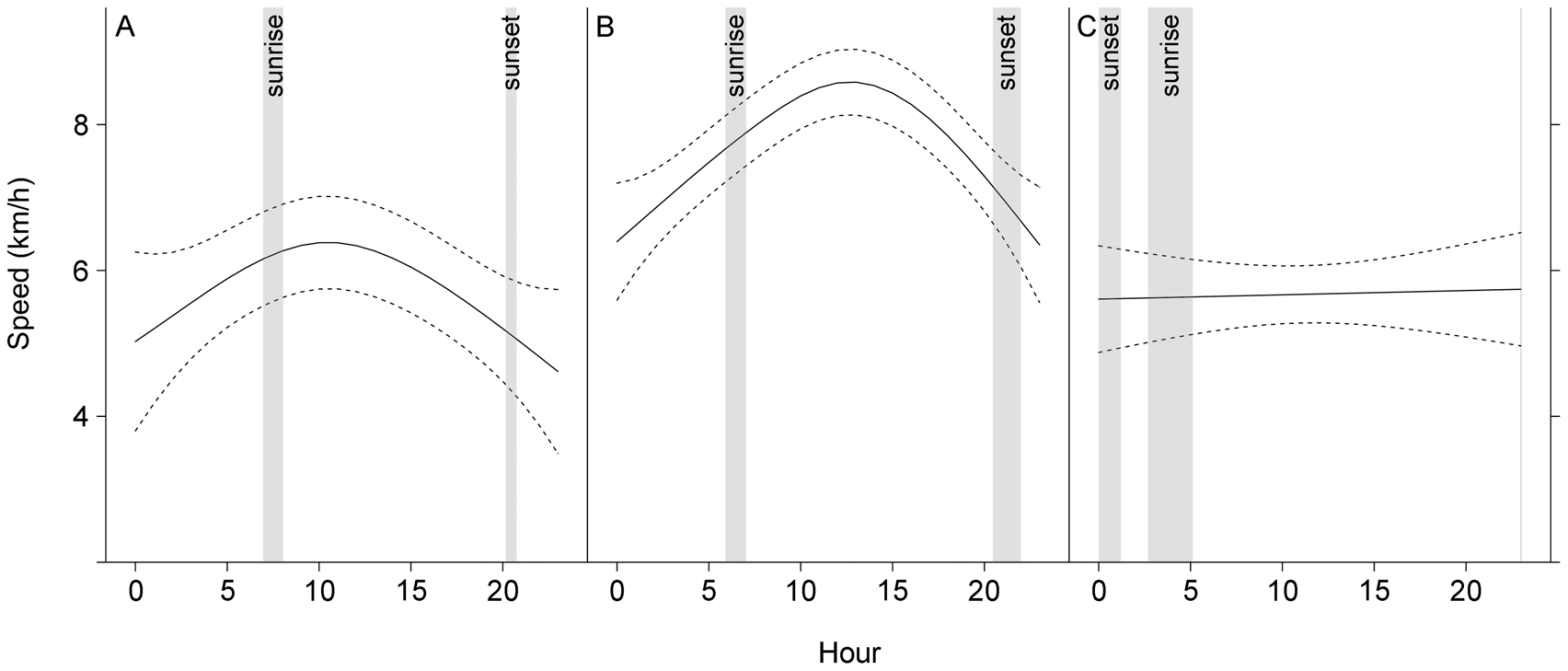
GAM estimate of transiting speed of fin whales during 24-hours in each geographic area. A. Azores area. B. area between 40 and 56°N. C. area north of 56°N. Solid line represents the estimate and dashed lines the estimate ±2 SE. Grey areas correspond to sunrise and sunset intervals (mean ± SD).

Time of day had a strong influence on transiting speed but the best fitting model had a different smooth function for each geographic area implying that the relationship differed among sites (Table S3). To understand how activity patterns of whales varied in relation to the day-night cycle in each geographic area, we calculated local sunrise and sunset times using the NOAA Solar calculator (http://www.esrl.noaa.gov/gmd/grad/solcalc/) and presented these along with model estimates of whales’ speed over a 24-h period (Fig. 5). South of 56°N, speed of whales increased gradually during the night, peaked from mid-morning to early afternoon (11–14 h) and declined to a minimum in the late evening. North of 56° the sun never dropped 12° below the horizon meaning that the period following sunset and preceding sunrise was of incomplete darkness. In this area, transiting speed remained unchanged throughout the day.

**Figure 6:**
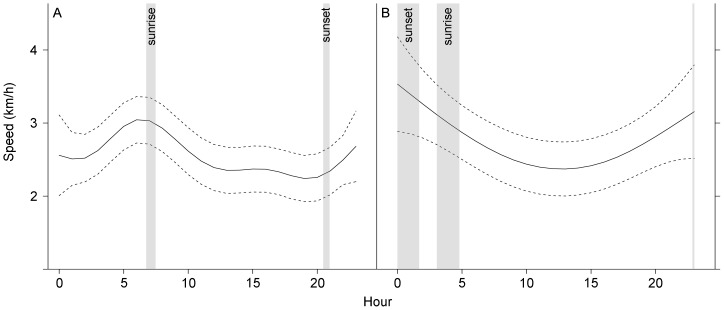
GAM estimate of ARS speed of fin whales during 24-hours in each geographic area. A. Azores area. B. area north of 56°N. Solid line represents the estimate and dashed lines the estimate ±2 SE. Grey areas correspond to sunrise and sunset intervals (mean ± SD).

We found no evidence of a daily cycle in ARS behaviour in the Azores (GLMM: t = −0.147, *P* = 0.883) or north of 56° (GLM: t = 0.20, p = 0.841) but speed of whales engaged in ARS varied significantly over time, with the highest speeds occurring between sunset and sunrise and the lowest from mid-morning to late afternoon in both areas (Fig. 6, Table S3). However, while in the Azores there was a clear peak in speed 1–2 h before sunrise, after which the speed decreased to increase again just prior to sunset, at northern latitudes the greatest speeds occurred around sunset. In contrast, sinuosity of whale tracks showed no relationship with hour of day (χ^2^ = 22.84, df = 23, p = 0.46) or geographic area (χ^2^ = 0.0005, df = 1, *P* = 0.980).

### Blue Whale Movements and Behaviour

**Figure 7:**
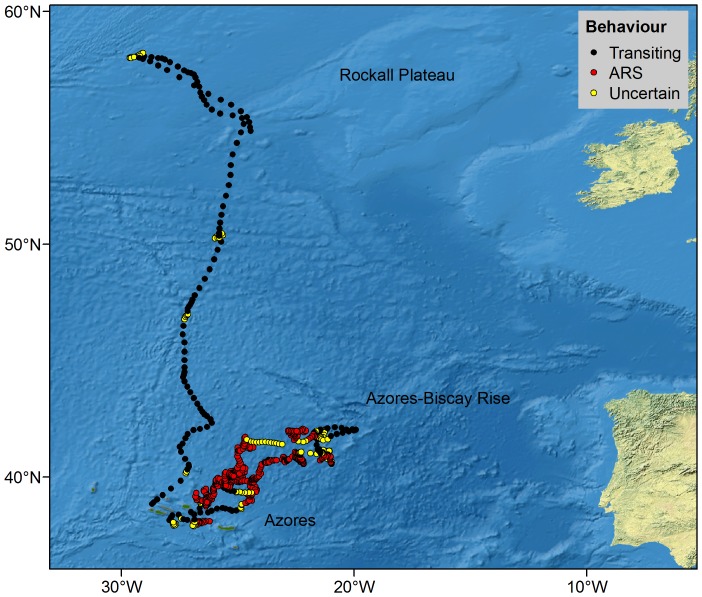
Hierarchical switching state-space model derived locations of blue whales showing inferred behavioural modes.

Whales 80709 and 80700 stayed within 800 km (highest latitude reached was 42°N) of the tagging area south of Pico Island for the duration of their tracking period (60 and 45 days, respectively) (Fig. 1, Table 2). Both whales headed eastwards in the first 2–4 days following tagging, switching to ARS as they reached a shallow hydrothermal vent (D. João de Castro) located between the central and eastern islands, and then alternating between behaviours while slowly moving northeast of the islands towards the King’s Trough-Azores-Biscay Ridge (Fig. 7). Mean proportion of track time per day spent in ARS was 78% (±38%) (Table 2). A total of seven discrete ARS areas were observed and whales spent an average of 246 hours (range = 16–756) within each area.

**Figure 8:**
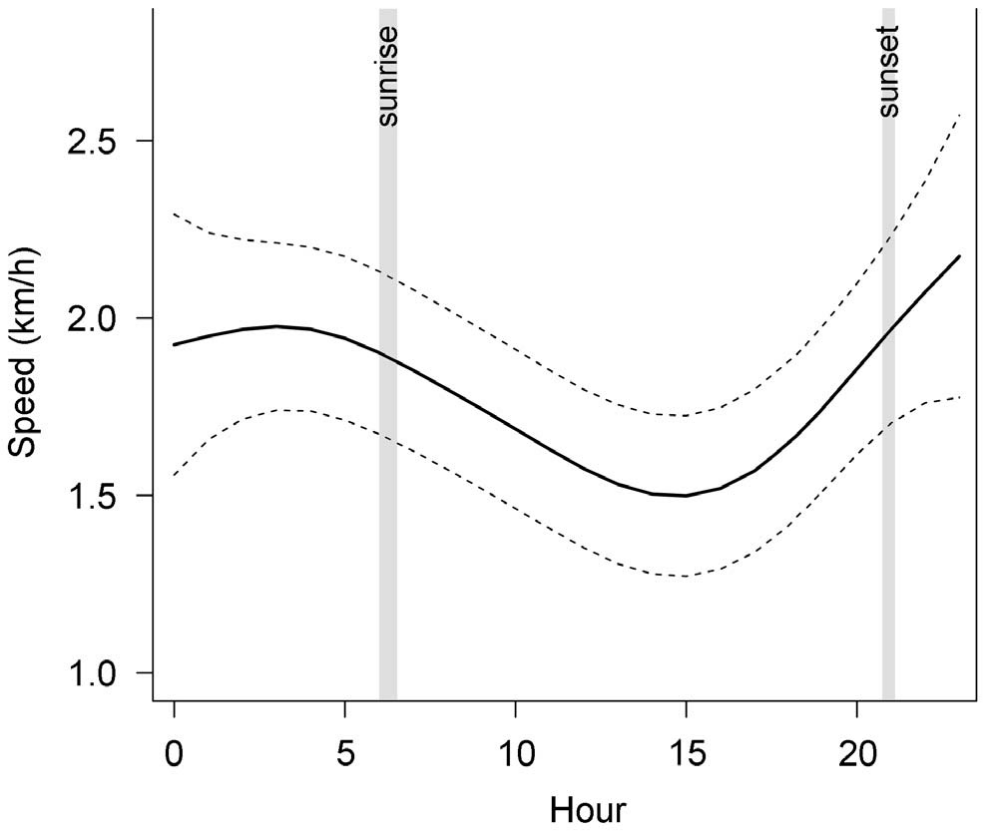
GAM estimate of ARS speed of blue whales during 24-hours in the Azores. Solid line represents the estimate and dashed lines the estimate ±2 SE. Grey areas correspond to sunrise and sunset intervals (mean ± SD).

Daily ARS patterns of blue whales were very similar to those of fin whales: probability of whales being in ARS (GLMM: t = −0.01, *P* = 0.524) and sinuosity of ARS paths (χ^2^ = 30.89, df = 23, p = 0.13) did not change over time but whales tended to move slightly faster from late evening to early morning with the lowest speeds around noon (GAM: smoother for hour: edf = 3.37, F = 2.83, *P* = 0.023; Fig. 8).

Whale 80718 departed from the Azores on the same day it was tagged. For 14 days it kept a northerly trajectory travelling over 2,000 km at an average speed of 6.5±3.7 km.h^−1^. Upon reaching the southern tip of the Rockall Plateau (55° 30′N), the whale turned northwest and slowed down (4.2±2.1 km.h^−1^). After 6 days around that area, the whale turned back to the Rockall Plateau, where the track terminated on 10 June (Fig. 7).

## Discussion

To the best of our knowledge, our study is the first to examine movements and behaviour of North Atlantic blue and fin whales during their spring migration. Despite a small sample size, the results presented provide important insights into the migratory strategies of these species, as well as novel information on their routes and destinations. Furthermore, the high temporal resolution of location data analysed in this study enabled us to document whales’ movements and behaviour with much finer detail than typically achieved in cetacean satellite telemetry studies.

### Whale Behaviour and Movements at Migratory Habitats

The fact that primary productivity and zooplankton abundance are considerably lower throughout the region south of the Subpolar Front Zone (48–52°N) [37], [38] has led to a general perception that biological productivity at these mid-ocean areas is insufficient to meet the high prey requirements of large rorquals. However, elevated phytoplankton and zooplankton biomasses have been found associated with specific oceanographic processes (e.g. the Azores Front (30–35°N, 30–34°W) [39], [40] and topographic features (e.g. seamounts [40]). Also, measurements of euphausiid density at discrete locations along the MAR revealed that mean density at some of the stations immediately north of the Azores (41–42°N) was not different from that found at other areas further north [41], [42]. Given a positive cost-benefit ratio, it is plausible that migrating whales will profit from exploring the enhanced productivity at discrete sites in mid-latitudes, prior to arriving at summering grounds. In the following lines we analyse the evidence in our data to support this supposition.

Baleen whales are capable of moving long-distances in short periods of time [18], [43], [44]. Although migrating blue and fin whales might have easily reached their summer ranges without major stops, except for one blue whale, all animals in this study paused their migratory journey and remained around the Azores from a few days up to two months. The estimated residency times provided in this study are bound to be underestimated, both because we have no way of establishing how long each whale may have already been in the area before tagging and because of the early termination of the tracks from 6 fin whales and 2 blue whales while the animals were still around the Azores.

Despite these shortcomings, our estimates of residence time at middle latitudes are far greater than previous ones based on photo-identification data (fin whales: 5 days, blue whales: 7 days [24]). Data from Visser et al. [24] were in many respects limited, having been collected across a restricted geographic area during vessel-based focal follows. Whales that were tagged in the study site of Visser et al. [24] moved far beyond the area and spent most of their track time outside it, explaining discrepancies between the two works in estimates of residency time and time spent foraging (see below).

While at middle latitudes, whales spent a substantial proportion of their daily time engaged in ARS behaviour (fin whales: 55%, blue whales: 78%). Although ARS may represent other behavioural states such as resting, breeding and other social interactions [45], we argue that, in this case, ARS essentially indicates foraging. First, tracking data were collected outside the known mating and calving seasons of these species [46]. Secondly, track sinuosity and repeated inshore-offshore movements detected in several tracks seem unlikely for resting or socializing whales. Thus, the majority of ARS behaviours should represent prey searching, although occasional periods of reduced activity and socializing behaviour are likely included. These results are in broad agreement (despite being more expressive) with those of Visser et al. [24], who reported that foraging activity comprised respectively 24% and 40% of the behavioural budgets of fin and blue whales seen in the Azores.

At sea observations of foraging activity and observation of faeces in the vicinity of tagged and non-tagged whales give further support to the idea that fin and blue whales feed in the area. Identification of prey remnants and analysis of stable isotopes from faecal samples revealed these whales preyed primarily on Northern krill (*Meganyctiphanes norvegica*) (unpublished data), in agreement with earlier observations in the area [25].

Another line of evidence for consistent foraging activity comes from the spatial and temporal distribution of ARS. To optimally exploit resources in patchy environments, foragers should intensively search in profitable patches while minimizing foraging activities in low-quality areas [28]. Thus, ARS behaviour is expected to occur in areas with high prey density. We did not attempt to correlate foraging effort of tagged whales with prey availability in the area, because existing data on the distribution of zooplankton are based on coarse surveys over broad spatial and temporal scales. Nonetheless, location of ARS behaviours strongly suggests that whales concentrated their foraging effort in areas where prey abundance is expected to be higher. Fin whale foraging locations were not widely spaced around the Azores, but instead there was a high degree of overlap between ARS areas. ARS locations were concentrated around the islands and over the MAR, north of the Azores. Consistent use of the area south of the islands of Faial and Pico, irrespective of month and year, and the pendular movements between these islands and the offshore shallow banks, indicates a high reliance of fin whales on this discrete site, possibly linked to its unique topography. Complex bottom topography and presence of isolated topographic features (e.g. islands, seamounts and submerged banks) tend to generate or intensify local physical processes that may result in enhanced primary production or in the retention and accumulation of phytoplankton and zooplankton biomass [47]–[49]. Little is known about the bio-physical processes in the vicinity of the banks and islands of the Azores but studies conducted at the MAR showed that increased vertical mixing and turbulence along the ridge are likely responsible for the higher biological productivity observed [50]–[52]. Island shelves and banks may also contribute to the retention of prey at shallower depths, facilitating prey capture [53], [54].

Foraging at middle latitudes was also inferred for two out of three blue whales tracked. Interestingly, ARS locations of blue whales did not match those of fin whales, even though individuals of both species were tagged in the same area. Also, ARS areas of the two blue whales occupied a wider area (extending from the islands to the Azores-Biscay Rise) and ARS behaviour occurred as whales progressed slowly to northeast. Despite these dissimilarities, data are clearly insufficient to draw any conclusions about differences in foraging strategies between the two species.

Track data for blue whales were insufficient to examine differences in foraging effort between months, but both whales spent a high proportion of time in ARS in May (85%) and June (74%). Fin whales spent considerably more hours foraging in April and May than in March and September. These results should be interpreted with caution because tracking data from the latter months came from only three individuals. Additionally, our analysis does not account for between-year differences in monthly variation in ARS behaviour, as tracking data from different years had to be pooled to increase sample size. Nonetheless, the overall picture of increased foraging effort in spring and early summer follows well with available, albeit limited, information on seasonal changes in the zooplankton community in the region. Zooplankton abundance and biomass tends to be higher between March and July, significantly decreasing towards September, with a slight smaller peak around October in some years [55].

We found considerable flexibility in residence time and time spent in ARS at middle latitudes between and within species. Assuming that whales primarily suspend their migration to replenish energy reserves, this is not surprising given that whales’ behaviour and residence should be influenced by a combination of exogenous (e.g. food availability, competitors density, environmental conditions) and endogenous factors (e.g. body fat condition, nutritional requirements (which in turn strongly depend on the age, sex and reproductive status of animals) and time programmes) [56]. Examining the effect and relative importance of each of these factors was beyond the scope of this study and will require larger sample sizes and collection of additional data.

Notwithstanding, some of our findings about residence time and ARS effort deserve to be further discussed. Our study suggests that fin whales tagged later in the year (weeks 20–36) tended to remain less time in the Azores. It is possible that, simply by chance, all whales tagged before week 20 were in the beginning of their residence period, whereas whales tagged after week 20 were close to the end of their residence period. Although this hypothesis cannot be ruled out, we find it unlikely given the marked difference in residence time for whales tagged before and after week 20 (Table 2). In addition, all tags that stopped transmitting before departure from the Azores were of whales tagged before week 20, meaning the tendency for decreased residence time with the progression of the year could be even more pronounced than our data suggest.

Length of fin whale residency at middle latitudes is expected to decrease if abundance of their prey declines below a given threshold reducing foraging efficiency. Lack of information about prey abundance in the area precludes direct investigation of this hypothesis. However, assuming ARS behaviour is a proxy for foraging effort, the number of days each whale spent at middle latitudes was independent from the proportion of time it was engaged in ARS. Moreover, decline of prey availability alone cannot explain why some of the whales tagged after week 20 initiated migration while whales tagged earlier were still foraging in the area. Fin whales 80704 and 89969, tagged on 12 May 2010 (week 20), spent 42% and 58%, respectively, of their time in ARS before resuming migration 6 and 4 days later. In the same period of time and area, whale 80707 (tagged in week 18) spent 96% of the track time in ARS. These findings suggest other factors aside from prey availability may influence the whales’ decision to depart from middle latitudes. The simplest explanation would be that whales tagged later foraged at other sites and already replenished some of the lost fat stores before coming to the Azores. However, it is noteworthy that, with the exception of the whale tagged in September, all fin whales that we documented departing from middle latitudes left the area in weeks 20–22. Whether this means that whales are responding to the same environmental cues (e.g. changes in photoperiod, water temperature) or internal schedules, or that departure from the area might be influenced by other whales’ decision to depart, remains unknown. Continuation of the telemetry study in the Azores might help elucidate migration schedules and some of the cues that trigger migration in these species.

### Migratory Routes and Destinations

Our study provides evidence of a central migratory corridor for fin whales in the North Atlantic and for the first time demonstrates a link between whales seen in the Azores in spring and summer and those found in the eastern Greenland and western Iceland feeding grounds [57]. Although only two fin whales were tracked to the feeding sites, another four whales were following the same trajectory when the tag stopped transmitting, suggesting a similar destination.

The two fin whales tracked to higher latitudes completed their migratory journey to the Irminger Sea-Denmark Strait in approximately 3 weeks travelling on average 135 km each day. Although the two whales differed on the path and destination (when they reached the Irminger Sea they were 750 km apart), they arrived within a few days of each other (3 and 6 June). Their arrival time agrees well with information from sighting surveys conducted in the region that show that fin whale abundance in the area starts increasing in the beginning of June, reaching a peak from mid-June to the end of July [58]. The northward pathway of the fin whale tagged in early autumn and tracked until 19 September is somewhat surprising. This raises the possibility that not all fin whales reach the high-latitude feeding grounds or that some whales may only arrive there in late autumn.

When tags deployed on blue whales stopped transmitting (10, 20 and 26 June) the three individuals were still far from their presumed northern feeding sites, suggesting that blue whales may arrive at high-latitudes later than fin whales. Sightings of blue whales west and southwest of Iceland peaked from mid-July to the end of August, representing a delay of approximately 1 month in relation to fin whale observations [58].

### Migratory Strategy of Fin and Blue Whales

Our results show that North Atlantic blue and fin whales forage during migration, contradicting the “feast and famine” paradigm of baleen whale migration but in line with other recent studies of blue whale migratory behaviour [17]. Long-distance migration is energetically demanding and reserves to be used for migration are a critical factor for most migrants [59]. Although cost of locomotion is lower for marine than terrestrial animals and tends to decrease with body size [60], even large-bodied animals like fin and blue whales must cope with the energetic requirements of a long migration. This is especially true during the northern migration, as whales have already used a significant part of the stored energy from the previous summer during the southbound journey and for reproductive activities.

Migrants use a variety of strategies to meet the energetic requirements of a long migratory journey. Some migrants adjust their migratory movements to track food resources over time. For example, ungulates and aquatic birds are able to pursue the phenological gradient of plant development towards their migratory destination, in order to have prolonged access to higher-quality forage [61], [62]. A similar strategy has been hypothesized for migratory baleen whales. The northbound movements of blue whales summering off southern and central California seems to match the northward progression of the bloom of primary production [63]. Visser et al. [24] reported that the timing of baleen whale occurrence in the Azores appears to follow the onset of the phytoplankton spring bloom by 3–4 months, which could indicate that baleen whales synchronize their migration to the North Atlantic phytoplankton spring bloom.

We found no evidence of ARS behaviour for fin whales migrating between the Azores and higher latitudes. Fin whales moved between these distant sites (over 3,000 km) along a nearly direct path, with no apparent detours to other areas, and spending little time en route. This is not consistent with the hypothesis of migrating fin whales “surfing the spring bloom” to continuously exploit the wave of secondary productivity that follows on. There are several reasons why tracking the prey bloom may be difficult or even unfeasible. The time lag between primary productivity and the higher trophic levels on which the whales feed likely varies greatly with local conditions (e.g. intensity of the primary bloom, environmental conditions and physical forcing mechanisms [64], [65]) creating discontinuities in prey development and increasing the chances for temporal mismatches between resource availability and whale migration. Moreover, even if the passage of the spring bloom results in elevated prey biomasses, absence of certain bio-physical processes that aggregate prey may render foraging ineffective in most open-ocean areas along the whales’ migratory path.

Prolonged residency of fin whales at middle latitudes, similarity in the behaviour of whales in the Azores and at northern latitudes, and the evident contrast of movement patterns observed in the former areas and during transiting to higher latitudes, suggest these whales may alternate long periods of active migration with sustained high travel rates with periods of extended use of specific habitats along the migratory route. Use of discrete stopover sites where animals can renew energy reserves and rest is a common behavioural trait among marine and terrestrial distant migrants [66]. Stopover sites should ensure access to plentiful food resources compared to other areas along the migratory path. Whales feeding on patchily distributed resources may orientate to habitats where they expect foraging success to be higher and intensify search effort there. If abundant prey patches are located, whales may remain in these discrete areas for extended periods of time, as seen for fin whales tracked around the Azores. How they locate these areas is currently uncertain but whales may be able to use distant cues (e.g. acoustic, chemical) to detect habitat features and processes that likely concentrate prey, use cues (e.g. acoustic) from other whales already present in the area or possibly retain memory of areas where they had greater foraging success in the past [67].

Usage of stopover sites doesn’t mean that whales cannot take advantage of occasional prey patches encountered during migration. Although we found no signs of whales interrupting their migratory progression to forage, short foraging bouts may have gone undetected in the analysis.

The migratory tactic of tagged blue whales was less clear. Movements and behaviour of the blue whale tracked to higher latitudes were similar to those recorded during the active migration of fin whales. But rather than focusing their foraging effort in approximately the same area as fin whales did, foraging blue whales gradually moved northwards, which could indicate they were trying to take advantage of the progression of the secondary productivity. Possible differences in migratory strategies and behaviour between the two species deserve to be further investigated, as it could help our understanding of how each species might respond to environmental changes arising from human activities.

Observations and molecular sexing (Silva and Prieto, unpublished data) of fin and blue whales seen foraging in the area, indicates that adult and subadult animals of both sexes may forage at stopovers during their northern migration. What proportion of the population uses this migratory strategy, under which circumstances, and to what extent these whales rely on mid-latitude foraging to complete their migratory journey, remain unclear.

### Diel Activity Rhythms

Despite the difficulty in interpreting 3-D animal movements using information from horizontal displacements measured at coarser temporal scales, satellite data strongly suggests that fin and blue whales exhibit diel rhythms in swimming activity. South of 56°N, fin whales in transit moved faster during sunlight hours than during darkness. In contrast, we found no evidence of diel cycles in transiting speed at higher latitudes where whales were exposed to constant light. This suggests that the increase in transiting speed at middle and intermediate latitudes may be related to visibility conditions. Marine mammals likely use a combination of sensory and environmental cues to assist them orienting, navigating over large distances with great precision, locating and capturing prey [43], [67]. Potential use of visual cues (e.g. sun position, presence of topographic features) to help in orientation and navigation might favour travelling at higher speeds during daylight hours. Whales may also adjust their swimming speed when travelling between prey hotspots or even during active migration to increase the chances of encountering patches of vertically migrating prey that might became available in surface waters at night. Under this scenario, it’s unclear why a similar pattern in swimming activity wasn’t evident in northern latitudes but differences in the pattern of diel vertical migration of zooplankton across geographic areas (see below) may help explaining variable results in fin whales’ transiting speed.

Satellite tracking data did not show a diel pattern of fin and blue whale foraging effort, as indicated by the occurrence of ARS irrespective from hour of day. However, we found a consistent diel signal in the swimming speed of whales in ARS, with enhanced speeds in late evening and early morning and decreased velocities following sunrise. Data on diel variations in swimming speed for large whales are scarce but those that exist are in disagreement with our findings and reported slightly higher speeds for fin whales during the day [68], [69]. It could be argued that the lower daytime ARS speeds in this study indicate periods of resting instead of foraging. However, the reduction in swimming speed was not accompanied by a change in track sinuosity as would be expected if whales were resting and showed only reduced levels of activity. Day-night differences in ARS speed may hint a behavioural response of the whales to the temporal dynamics of their prey. The timing of the peak in ARS swimming activity of fin and blue whales further suggests a relation to prey diel activity patterns. Three-dimensional analyses of diving whales combined with measurements of prey field are necessary to understand how these predators adjust their search strategies to prey movements. Until those data are available we can only speculate on the underlying reasons of the observed patterns.

At midlatitudes, the diel vertical migration (DVM) of zooplankton is closely synchronized with the day-night cycle, with the bulk of the biomass remaining at depth during daylight, ascending to near surface waters at dusk and migrating downwards just before sunrise [70]. Whales may intensify their foraging effort and became more active during the night to take advantage of the increased availability of prey in surface waters. Several studies demonstrated that the DVM behaviour of zooplankton persists even under the continuous illumination of the Arctic summer but migration towards the upper layers generally occurs later (around midnight), individuals remain there for less time, and the upward and downward motion is sometimes unsynchronized [71], [72]. This could explain the 2–3 h delay and the shorter duration of the peak in fin whales’ ARS speed at higher latitudes.

In addition to undertaking diel vertical migrations, krill swarms occur in dense aggregation during the day to avoid predation and disperse at night to facilitate feeding on phytoplankton [70]. Consequently, whales foraging at night may need to cover larger distances to cope with overdispersed prey, which would result in an apparent increase in their horizontal speed. Finally, recent studies showed that the horizontal swimming speed of krill at night and dusk (during the vertical ascent) was more than twice their diurnal average speed [73], [74]. Coupled with other escape responses, such increase in krill’s speed might force whales to increase their pursuit and/or lunging speed.

### Conclusions

Migration is an important, but often overlooked life-history component of large baleen whales, partly because of the difficulty of studying the daily routines of these whales over much of their ranges and throughout their annual cycles. By instrumenting migrating blue and fin whales with satellite transmitters, we were able to reveal several aspects of their behaviour with considerable detail. The aim of this study was not to produce a comprehensive description of the migratory behaviour of these species: a much larger dataset and a longer study period will be required for this. Additionally, the small sample size, especially with respect to blue whale data, limited interpretation of the data and prevented inferences at the population level. Despite these caveats, our results offer new insights into baleen whale migration and prompt interesting hypotheses that are worth investigating.

Our study provides compelling evidence for mid-latitude foraging in central North Atlantic waters for fin and blue whales migrating to the northern feeding sites. More importantly, we show that these species can suspend their seasonal migration and remain foraging in middle latitude areas for extended periods of time and much later into the summer than generally assumed. Behaviour of fin whales during migration has some resemblances to that of several birds of prey and ungulates that rely on a series of stopover sites located along migration routes to accumulate energy reserves. If the use of stopover sites is a common strategy for large whales, habitat degradation or disturbance at these sites may reduce foraging opportunities for migrating whales. A better understanding of migratory behaviour is needed to assess if loss of stopover habitats can compromise the ability of whales to reach and successfully explore northern feeding grounds. Finally, our study provides the first empirical evidence for a migratory corridor for fin and blue whales located in the central North Atlantic and for a linkage between fin whales seen in the Azores and those found in eastern Greenland-western Iceland.

## Supporting Information

Figure S1 **Smooth estimate of Latitude in the GAM for fin whale transiting speed.** Dashed lines represent the 95% confidence intervals. Degrees of freedom are shown in parentheses.
(TIF)

Table S1 Posterior medians and 95% credible limits (CL) for movement parameters θ (turn angle) and γ (combined autocorrelation in direction and speed) estimated from the hSSSM models fit for fin and blue whales.
(DOCX)

Table S2 Summary of parameter estimates from generalized linear mixed model for proportion of tracking hours fin whales spent in ARS per day.
(DOCX)

Table S3 Summary of parameter estimates from generalized additive models for fin whale travel speed during transiting and ARS behaviours.
(DOCX)
