# Supplementary figures and images for: North Atlantic Blue and Fin Whales Suspend Their Spring Migration to Forage in Middle Latitudes: Building up Energy Reserves for the Journey?
Source: PLoS One. 2013 Oct 8;8(10):e76507. doi: 10.1371/journal.pone.0076507 (PMC3792998; doi:10.1371/journal.pone.0076507)

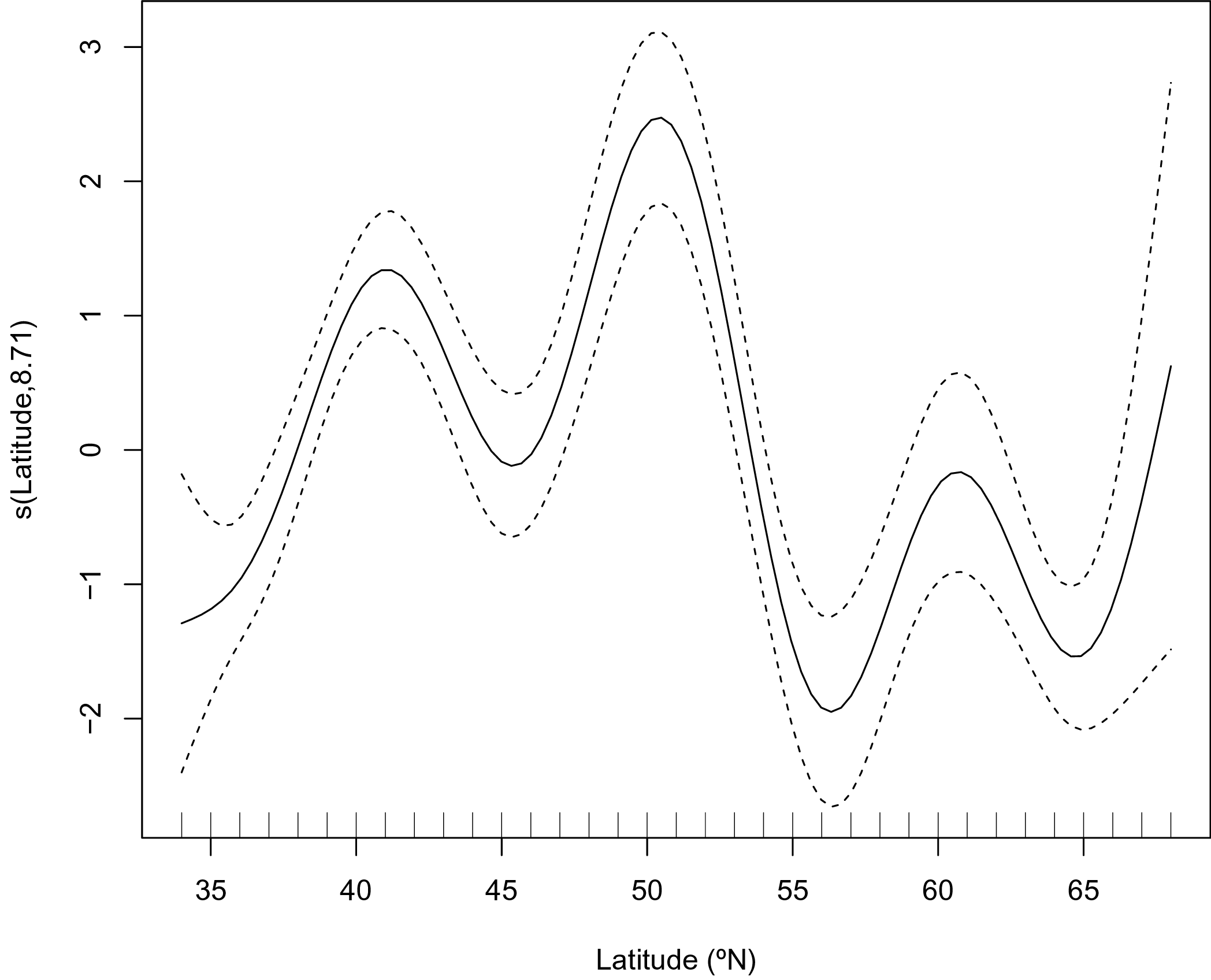

Supplement: Figure S1 — Smooth estimate of Latitude in the GAM for fin whale transiting speed. Dashed lines represent the 95% confidence intervals. Degrees of freedom are shown in parentheses. (TIF) [file pone.0076507.s001.tif]
