# Supplementary material for: North Atlantic Blue and Fin Whales Suspend Their Spring Migration to Forage in Middle Latitudes: Building up Energy Reserves for the Journey?
Source: PLoS One. 2013 Oct 8;8(10):e76507. doi: 10.1371/journal.pone.0076507 (PMC3792998; doi:10.1371/journal.pone.0076507)
Supplement: Table S1 — Posterior medians and 95% credible limits (CL) for movement parameters θ (turn angle) and γ (combined autocorrelation in direction and speed) estimated from the hSSSM models fit for fin and blue whales. (DOCX) [file pone.0076507.s002.docx]

|  | Fin whale model | | | Blue whale model | | |
| --- | --- | --- | --- | --- | --- | --- |
|  | Median | 2.5%CL | 97.5%CL | Median | 2.5%CL | 97.5%CL |
| θ*_transit_* | 3.8 | 0.2 | 8.7 | 3.1 | 2.7 | 3.5 |
| θ*_ARS_* | 105.3 | 154.7 | 194.8 | 177 | 155 | 201 |
| γ *_transit_* | 0.84 | 0.81 | 0.87 | 0.86 | 0.78 | 0.93 |
| γ *_ARS_* | 0.07 | 0 | 0.15 | 0.10 | 0.01 | 0.24 |
