# Supplementary material for: North Atlantic Blue and Fin Whales Suspend Their Spring Migration to Forage in Middle Latitudes: Building up Energy Reserves for the Journey?
Source: PLoS One. 2013 Oct 8;8(10):e76507. doi: 10.1371/journal.pone.0076507 (PMC3792998; doi:10.1371/journal.pone.0076507)
Supplement: Table S2 — Summary of parameter estimates from generalized linear mixed model for proportion of tracking hours fin whales spent in ARS per day. (DOCX) [file pone.0076507.s003.docx]

| Terms | Factor | Proportion time in ARS | | |
| --- | --- | --- | --- | --- |
|  |  | ß | SE | *P* |
| Intercept |  | -0.67 | 0.12 | <0.001 |
| Month^[[1]](#footnote-1)^: | April | 2.00 | 0.17 | <0.001 |
|  | May | 1.98 | 0.17 | <0.001 |
|  | September | -1.73 | 0.54 | 0.001 |

1. Reference level for Month: March.

   ß=Parameter estimate.

   SE=Standard error. [↑](#footnote-ref-1)
