# Supplementary material for: North Atlantic Blue and Fin Whales Suspend Their Spring Migration to Forage in Middle Latitudes: Building up Energy Reserves for the Journey?
Source: PLoS One. 2013 Oct 8;8(10):e76507. doi: 10.1371/journal.pone.0076507 (PMC3792998; doi:10.1371/journal.pone.0076507)
Supplement: Table S3 — Summary of parameter estimates from generalized additive models for fin whale travel speed during transiting and ARS behaviours. (DOCX) [file pone.0076507.s004.docx]

| Terms | Factors | Parametric coefficients | | | Non-parametric smoothers | |
| --- | --- | --- | --- | --- | --- | --- |
| Response: *Transiting speed* | | ß | SE | *P* | edf | *P* |
| Model 1 |  |  |  |  |  |  |
| Intercept |  | 6.76 | 0.09 | <0.001 |  |  |
| s(Latitude) |  |  |  |  | 8.71 | <0.001 |
|  |  |  |  |  |  |  |
| Model 2 |  |  |  |  |  |  |
| Intercept |  | 4.84 | 0.35 | <0.001 |  |  |
| Area^[[1]](#endnote-1)^: | 40–56ºN | 2.31 | 0.39 | <0.001 |  |  |
|  | >56ºN | 0.42 | 0.19 | 0.030 |  |  |
| s(Hour)×Area: | Azores |  |  |  | 2.88 | <0.001 |
|  | 40–56ºN |  |  |  | 3.25 | <0.001 |
|  | >56ºN |  |  |  | 1.42 | 0.392 |
|  |  |  |  |  |  |  |
| Response: *ARS speed* | |  |  |  |  |  |
| Intercept |  | 1.02 | 0.42 | 0.016 |  |  |
| s(Hour)×Area: | Azores |  |  |  | 6.39 | <0.001 |
|  | >56ºN |  |  |  | 2.90 | <0.001 |

1. Reference level for Area: Azores.

   ß=Parameter estimate.

   SE=Standard error.

   edf=effective degrees of freedom. [↑](#endnote-ref-1)
